# Student Subjectivity in the Marketised University

**DOI:** 10.3389/fpsyg.2021.827971

**Published:** 2022-02-28

**Authors:** Geoff Bunn, Susanne Langer, Nina K. Fellows

**Affiliations:** Department of Psychology, Manchester Metropolitan University, Manchester, United Kingdom

**Keywords:** student engagement, student agency, Lacanian analysis, the four discourses, the student journey, *objet petit a*, marketisation

## Abstract

We present data from an exploratory qualitative interview-based pedagogical research project on the development of student agency in higher education. Our aim was to respond to Nick Zepke’s claim that what is often missing from the current neoliberal discourse of higher education ‘is students having a voice in what and how they learn and how they can action their voice in the wider community as agentic citizens.’ Informed by Lacanian discourse analysis, our project investigated the opportunities and threats facing some of our undergraduate students as they struggled to exercise agency and develop autonomy in the marketised university. Repeat interviews (*n* = 15) with final year students focussed on the psychosocial categories of power, affect, intersubjectivity and desire. The analysis was guided by Lacan’s theory of the four discourses, an account of the vicissitudes of agency. We found that students can move between discourses depending on the extent to which their agency (operationalised here as Lacan’s ‘object cause of desire,’ the *objet petit a*) was enabled or thwarted. Our critique of the metaphor of the ‘student journey’ addresses the implications for learning and teaching and the university’s mission to develop its students in light of perceived commercial pressures.

## Introduction

Increasingly informed by neoliberal ideology, the global higher education sector is being profoundly reshaped by a combination of information technology and the rhetoric of economic accountability to develop entrepreneurial, global-facing, market-oriented universities (McGettigan, 2013; Clarke, 2019). National and international league tables, expensive and prestigious building projects, research assessment exercises and other putative indicators of excellence inform and enact institutional policies and practices (John and Fanghanel, 2016). One key problem, however, is that metrication systems designed to afford and measure returns on their investments for students, parents, and universities alike are eroding the long-standing traditional ambitions of higher education to produce well-educated and ethically informed citizens (Molesworth et al., 2011; Nixon et al., 2016). As education becomes increasingly commodified, the acquisition of critical citizenry skills becomes incidental ‘to an imperative to maximise one’s human capital in a ruthlessly competitive labour market’ (Kilner et al., 2019, pp. 111–112). The result is the introduction of teaching quality assessment devices and student satisfaction surveys (e.g., the National Student Survey [NSS] in the United Kingdom) and the ‘Teaching Excellence Framework’.^1^ Such instruments are designed to facilitate competitiveness between institutions and fabricate a so-called free-market in order to sanction increasing tuition fees and engender market exit for failing institutions. A rhetoric of student-centred provision accompanies such innovations, together with an avowed emphasis on social mobility and consumer choice (Gourlay and Stevenson, 2017).

Students have been led to believe that a bright future awaits them at the terminus of their journey. Universities consequently endeavour to nurture their students’ curiosity, skills and ambition. Our institution for example is committed to developing our students’ “intellectual powers, creativity, independent judgement, critical self-awareness, imagination, and personal skills that will clearly identify them as global learners [and] world class professionals” (MMU SLTA 2.3). Such aspirations inform and underpin learning and teaching principles in many institutions across the sector. Nevertheless, many conventional higher education teaching practices are frequently at odds with such well-meaning objectives. Some of the problems experienced by students are caused by ‘the cultural values and assumptions which underpin different aspects of pedagogy and assessment’ (Case, 2015, citing Haggis, 2006, p. 533), including, as many have argued, the neoliberal marketisation of the sector (Collini, 2012).

As Jennifer Case (2015) reminds us, our goal as educators should be to produce students who ‘leave higher education with different knowledge and capacity for action than that with which they entered.’ Acknowledging the ontological turn in student learning research, which shifted attention away from knowledge and skills towards personhood, being and becoming, Case argues that becoming an active agent (a process she calls ‘agential morphogenesis’) involves the simultaneous transformation of personal and social identity. Investigating innovative ways to conceptualise and generate agency must therefore become a priority for all stakeholders in the university, from administrators to academics and, of course, to students themselves. Student satisfaction, engagement and retention, together with discourses around employability and lifelong learning have encouraged a renewed focus on agency as a key component of student success. However, student agency has (paradoxically) become a target of knowledge to be operationalised, nurtured and developed by mechanisms of power aligned with neoliberal ideas about what the business of education should be (Zepke, 2018). The result is the imprinting of agency with an ideologically shaped and monolithic meaning (Hethrington, 2015). We oppose this development and wish to reclaim student agency as a multi-faceted intersubjective function that is enabled or thwarted by networks of power and therefore cannot be operationalised in terms of a presence/absence deficit model (as in ‘resilience’ discourses for example, see Webster and Rivers, 2019). We do so by drawing on Lacan’s theory of discourse applied to higher education (Johnson, 2014).

### The Metaphor of the Student Journey

Nick Zepke (2015) argues that despite its complexities, student engagement can be conceptualised as taking either traditional or progressive forms. Conventionally, engagement can be characterised pragmatically, oriented towards the sequential acquisition of employability skills. Or, more progressively, it can be thought of ‘as a social–cultural ecosystem in which engagement is the glue linking classroom, personal background and the wider community.’ Mainstream research on student engagement, which tends to operationalise teaching and learning as quantifiable and auditable, demonstrates an elective affinity with neoliberalism’s stipulations that higher education must increase the student’s economic value by conveying practical knowledge, building performativity and assuring quality through accountability (Zepke, 2018). In the alternative emancipatory paradigm, however, engagement research focuses on knowledge and ethical virtues. The aim is to develop ‘a democratic–critical conception of learning that is participatory and dialogic, leading not only to academic success but also to success as citizens.’ What is often missing from the contemporary neoliberal discourse of higher education, Zepke argues ‘is students having a voice in what and how they learn and how they can action their voice in the wider community as agentic citizens.’ (Zepke, 2018, p. 441).

Our ambition in this paper is to respond directly to this appeal by presenting data from a psychosocial research project that had engagement as one of its foci. One of the aims of our project was to problematise the conventional view of student agency in terms of a deficiency model whereby the student journey is assumed to complete what is lacking in the novice student. In our view, the metaphor of the student journey functions more as a sort of conveyor belt for the acquisition of predictable skills rather than as a potentially perilous expedition or quest. This master signifier is an example of what Zepke refers to as a ‘fixed and generic engagement framework…used to enable compliant students to persist, improve achievement, graduation and employment’ (Zepke, 2018, p. 439). But definitions of success, he reminds us, ‘do not have to be limited to persistence, passing courses, graduating and gaining employment.’ Derived from a pernicious consumer rationale, the metaphor of the student journey implies that attaining a degree is merely a matter of moving in a straight line from beginning to end, picking up the requisite skills in the right order and not inadvertently wandering down any apparently unproductive or potentially dangerous dead ends. This generative metaphor (Danziger, 1990) propagates normative aspirations of unproblematic belonging, validated and immediately useful achievements, and predictable and measurable outcomes. Under this framework, students start out on their university education as ‘empty vessels’ lacking in knowledge, skills and agency, to be passively filled up as they move along in space and time (Freire, 1970). The journey metaphor is limited precisely because it possesses a minimal sense of struggle, few genuine hazards to negotiate, and no potential for failure (the latter arguably a key factor in learning). Nor does it consider student individuality. A more effective account of agency, we will argue, would not only be personally meaningful to students themselves but would also contain a necessarily disruptive element. Like terms such as ‘democracy’ or ‘freedom,’ the empty master signifier of the ‘student journey’ appears to contain something more than itself, a ‘surplus of meaning which we attempt to capture in our struggles to define’ (Brown et al., 2006, p. 130).

In our formulation, that elusive surplus of meaning is the student’s *objet petit a*, a perceived lack that is therefore their object cause of desire. An elusive and paradoxical concept in Lacanian theory, the *objet a* is related to the formation of the subject’s identity and experience.^2^ The “lack of being that causes all desire” according to Bracher (1994, p. 114), the *objet a* is the radical unbridgeable gap that expresses the subject’s incompleteness and, as a result, constitutes their desire. According to Richard Boothby, the *objet a* is a liminal aspect of the psyche, “strangely suspended between the subject and the other, belonging to both and neither” (Boothby, 2001, p. 243). In terms of the development of student agency, the *objet a* is a relational category that encapsulates both the exhilaration of absolute possibility and the comfort of realistic contingency. A notoriously intangible and paradoxical entity, the *objet a* as an outrageous amalgam of desire and ideology, a generative lack or constitutive void. For our purposes, we are (dis)content to conceptualise the *objet a* as a metonym for agency that has both a phenomenological and an ideological presence (Žižek, 1989).

### Towards a Psychosocial Pedagogy

Our study aims to contribute to a *psychosocial* approach to pedagogical research by examining the ways in which educational experiences are entangled with social, cultural, and political forces (S. Clarke, 2002; M. Clarke, 2019). Drawing inspiration from sociology, Freudian and Lacanian psychoanalysis, critical theory and post-structuralism, psychosocial research is intrinsically interdisciplinary (Parker, 2010). Opposing the objectification and reification of the human subject (Frosh, 2003) and adopting a non-positivistic epistemology and a non-reductionist ontology, psychosocial research orientates towards progressive political, social, and personal change. We hope to contribute to theorising student subjectivity in the era of marketisation of higher education.

Lacanian psychoanalysis in general, and Lacan’s discourse theory in particular, has had a significant influence on educational theory and policy (Brown et al., 2006; Roseboro, 2008; Clarke, 2019). Lacan’s theory of the four discourses is an attempt to understand how different interpersonal relationships can generate different forms of subjectivity (Bracher, 1994, p. 107; Lacan, 2007; Clarke, 2019). The discourses are theorised to have different phenomenological consequences for those caught up in them. The theory formulates the complexities of the intersubjective matrix as a social nexus that accounts for how speaking, listening and acting generate systems of meaning “that define things for people and define people for other people” (Parker, 2010, p. 29). Although the four discourses represent four differentiated interpersonal configurations, each one is structured around a speaking *agent* and a listening *other*. In our formulation, whereas the discourses of the master and the university correspond to Zepke’s mainstream modes of student engagement, the discourses of the hysteric and the analyst correspond to Zepke’s emancipatory paradigm.^3^ As a mobile, dynamic signifier, the *objet a* plays a different (but fundamental) role in each of the four discourses.

Two of the four discourses (those of ‘the master’ and ‘the university’) produce impoverished modes of student agency, we argue, despite being the most prevalent discourses in contemporary higher education. According to Lacan, the Master’s discourse is the discourse of capitalism, the elucidation of which is the function of University discourse (Clarke, 2019). The master addresses the student as an apprentice, at best, or at worst, as a slave. The students work for the lecturer, not themselves, by validating the lecturer’s desire. University discourse valorises bureaucracy: assessment deadlines, attendance monitoring, matriculation rules, audit processes, and so on. Agency is only permitted to act within these narrow parameters. Through no fault of their own, students who remain trapped within university discourse inevitably fall short of the institution’s stated ambitions for them.

Master and university discourses can be understood as imperialising projects, where difference must yield to the production of surrogate identifications (Brown et al., 2006, p. 131). From the vantage point of the student’s positioning, the master’s discourse produces Subservient Agency (the student works for the lecturer’s enjoyment) whereas university discourse produces Subsistence Agency (the student is ‘making do and getting by,’ see Table 1). Both are exemplars of Zepke’s (2018) ‘mainstream student engagement.’ Zepke argues that student agency can be understood not only in terms of conventional values of knowledge and skills on the one hand, but also critically, in relation to the quest for meaning, ethical purpose and citizenship on the other. Building on this distinction, we predict that critical forms of agency are nurtured by the two marginalised discourses: those of the hysteric ($) and the analyst (a). Although these have the greatest potential for agentic development, we argue that they are marginalised in contemporary higher education. They are insufficiently nurtured, we claim, because the neo-liberal university considers them to be maverick, disruptive or expensive (Clarke, 2019). A critical model of student engagement, we argue, must nevertheless include these two forms of agency namely, Subliminal Agency and Sublime Agency, respectively. Mainstream pedagogy usually silences or pathologises hysteric’s discourse thereby thwarting the emergence of Subliminal agency. Although Sublime Agency is the most effective and desirable form, it is also the most precarious (if not downright risky) mode of subjectivity in the current era.

**TABLE 1:** Four modes of student agency.

	Subservient agency	Subsistence agency	Subliminal agency	Sublime agency
Lacanian Discourse	master	university	hysteric	analyst
Function	governing, hegemony	educating, interpellation	protesting, resistance	revolutionising, change
Lacanian Matheme (student in bold)	S1 → **S2** $ // a	S2 → **a** S1 // $	**$** → S1 a // S2	**a** → $ S2 // S1
The student is positioned as…	S2, the recipient of and responder to the lecturer’s (S1) demands.	The passive *objet a*, hystericised by the university’s (S2) bureaucratic demands.	$, a divided subject, producing oppositional outbursts against the university (S1).	an active *objet a*, motivated by their own desire, addressing contradictions ($).
Fate of the student’s *objet a*	The master (S1) enjoys the student’s production of the *objet a.*	The university (S2) defines a proxy *objet a* for the student.	The anxious student ($) is motivated by but is unaware of their *objet a*.	Supported by knowledge (S2), the student embodies their *objet a*.
Our students’ **major** (and minor) roles	(Jack)	**Jack** **Caitlin**	**Mae** **Zach** (Caitlin) (Molly)	**Molly** (Mae) (Zach)

*The discourses framing student agency have profound implications for student learning. Bold highlight indicates the position occupied by the student in each matheme. Sources: Bracher (1994), Brown et al. (2006).*

Mann (2001) argued that many student learners adopt either a *surface approach* to their studies (rote learning, unreflective reproduction of material, task-focussed orientation and so on, operating as the addressee in master’s discourse), or a *strategic approach* (a focus on meeting assessment requirements and lecturer expectations and securing high grades, operating as the addressee in university discourse). Both approaches rely on impoverished opportunities for the exercise of agency precisely because they locate the responsibility for success in the perceived desires and demands of ‘the other,’ not the self. In Lacanian terms, surface learning occurs when the student (S2) is addressed by the lecturer speaking from the master’s position of authority (S1). The student works for the lecturer, perversely, as the slave works for the master.^4^ The student’s agency is defined by the lecturer/master who secretly enjoys receiving the gifts of the student’s creativity (*objet a*). Strategic learning arises when the bureaucratic demands of university discourse (S2) traps the student (now positioned as a raw and uncultivated *objet a*) into an ethic of “performativity and functionality; a greater focus on efficiency and effectiveness at the expense of complexity and ambiguity…and especially the educational life course, as institutionalised, following normatively and inexorably the same ‘prescribed’ path” (Mann, 2001).

## Materials and Methods

Our project was an exploratory qualitative interview-based pedagogical study on the development of student agency in higher education. The research adhered to the British Psychological Society’s Code of Ethics (British Psychological Society, 2018) and Ethics approval was granted by our Faculty Ethics Committee. As teachers and researchers, we were committed to ensuring that students did not feel pressured to participate so we (GB and SL) removed ourselves from the interview process. The research was introduced and advertised in class, and the project’s research assistant (NKF) coordinated recruitment, conducted the interviews, and collected the data. Participants consisted of final year undergraduates taking a critical psychology course, a popular, interdisciplinary, and interactive unit that makes explicit the concepts, paradigms and power structures that frame the discipline. Students were invited to participate by email during the autumn and then again in spring. Recruitment resulted in nine initial interviews, with a further six follow-up interviews (*n* = 15). Two of the nine participants were male. Participation was rewarded with the standard rate of psychology participation pool credits which can be redeemed by students for their own research. Interviews were conducted using a flexible, semi-structured topic guide that explored instances of the four discourses in students’ university lives. The interview data was transcribed and anonymised.

Lacanian discourse analysis guided our transcript analysis (Parker, 2010; Neill, 2013). We first read each transcript without making any systematic interpretations. A second individual reading then engaged with the text more abstractly, noting not only subject positions, master signifiers and moments of transition, but also rhetorical features such as metaphor, storytelling and patterns of diction. The principal researchers (SL and GB) subsequently discussed each transcript extensively. Collaborative analysis challenged individual interpretations and added new layers of insight and meaning. Differences of interpretation were negotiated through dialogue. We also continuously updated a meta-analysis log, to record further observations and theory-building ideas. Discussions with our researcher (NKF) further helped to triangulate and consolidate our interpretations. A series of vignettes of all six repeat interviewees concluded this preliminary stage of the research process.

### Results: Five Student Vignettes

We have used extracts from four of these vignettes to evidence the ways in which students navigate their own journeys. Our aim is to highlight how the relationship between interpersonal situations and intrapersonal forces enable different forms of agency. We focus on two salient aspects of their journey: the students’ engagement with their final year dissertation project and the object cause of desire, the *objet a*. The dissertation was a large piece of independent empirical research that constituted a quarter of the students’ final year mark. It is an essential precondition for eventually achieving professional recognition. The most eligible dissertations are published in the Department’s e-space repository and can count as a first publication. This significant piece of work was sometimes aligned with a student’s *objet a* but not always.

### Jack’s Journey

Jack vacillated primarily between the discourses of the master and the university, that is, between exercising subservient and subsistence agency. The son of a retired policeman whose advice he took in choosing to study Psychology, Jack happily adopted the role of the obedient apprentice. He employed a highly methodical study regimen, extolled the virtues of self-discipline, and viewed the university in almost Darwinian terms: it “whittles out the weak,” he said (J1 256). He positively endorsed the stressful consequences of university discourse, accepting anxiety as both a necessary challenge of student life and as a disciplinary force: “it teaches you how to control your emotions, your time, your work ethic,” he said, “all them things which I think you need to then move on to a job or whatever…I think you do need a bit of stress in university.” [J1 249–254].

In deferring to the university’s authority and adopting a carefully systematised work schedule, Jack sought to suppress the anxiety produced by the confrontation of the those demands with his attributed deficiencies, namely, his difficulties with spelling and grammar, and answering open-ended questions (J1 134–144, 155–159). He was unflustered by assessment deadlines, word limits, and attendance monitoring, and exams were his preferred form of assessment. He appreciated Multiple Choice Tests for their predictability and sense of order. “I’m not very good at writing essays,” he confessed, “but hopefully the quality’s there. Like I’ve still got another 15 days, 14 days left, and I’ve got another two thousand words left, so it should be - it should be fine.” (J2 190–192) Jack was satisfied with his methodical approach: “once you’ve ticked everything off your list you feel good about yourself,” he said (J2 202–203). A self-controlled student who imposed routines on himself, Jack planned his assignments conscientiously and in good time and prepared for lectures in advance. Although he said he rarely answered questions in large lectures, he appreciated being recognised by his lecturers, but only for strategic reasons, not in order to forge a nurturing interpersonal connection. Speaking from a position of subsistence agency, he claimed he wouldn’t normally do any extra reading unless it was for a specific purpose such as preparing for an exam. When asked how he thought the university saw him, he replied: “I wouldn’t say I’m even on the radar…just because – I don’t know, I’m not a standout student.” [J1 189–190].

The uncharted territory of the dissertation was a considerable source of anxiety for Jack. He felt insufficiently prepared for this large piece of independent research and initially described himself as “still floating in the water” [J1 362]. His unease was further exacerbated by having an apparently indifferent and unresponsive supervisor. Realising this was inducing more stress than it was worth, Jack “lost faith” and decided to “try it off his back” [J2 203, 206]. By the time of our second interview, this strategy had enabled him to conquer any rising panic and settle back into his subservient comfort zone. The imposition of order came at the expense of forsaking the pleasure inherent in the pursuit of the *objet a* as part of the dissertation. Instead of affording an opportunity for self-directed learning and growth, the dissertation became merely a manageable chore. This was a price Jack was willing to pay, though, because his *objet a* was resolutely located beyond the university. He wanted to get a job in a shop, learn a foreign language and go travelling with his new girlfriend. Together with the prospect of earning decent money and taking up rugby again, his plan propelled him through the slog of his final year. As his non-academic ambitions started to coalesce, the proxy *objet a* conferred on him by the university began to diffuse. By the end of the interview, Jack was looking forward to the pleasures of “real life” [J2 333–334]. “I’ve had enough of education” he concluded, “twenty-one years has been enough.” [J2 101].

### Caitlin’s Journey

Caitlin valued professionalism and positioned herself beyond master’s discourse, oscillating instead between the discourses of the university and the hysteric. A mature student who had worked since she was 14, Caitlin didn’t appreciate what she considered being patronised, taught like a school pupil, or attending badly organised seminars. She enjoyed class discussions when they provided valuable learning experiences that went beyond what the lecturer had written on the slides. She appreciated being recognised as an individual by lecturers and didn’t want to be just a face in the crowd. Motivated by both professional advancement and personal fulfilment, she was aiming to secure “that line on my CV that says ‘Degree in Psychology”’ [C1 297].

Caitlin saw university as analogous to the world of work, which had taught her the subsistence skills of meeting deadlines and following rules. “I’m a mature student,” she said, “so I’m older and I’ve worked and know obviously about hitting deadlines and things like that but obviously the majority of people here are younger, so obviously it teaches them what it’s like.” [C1 171–173] She enjoyed conducting independent research motivated by her own curiosity and could easily think of examples of work she had produced that she was proud of and inspired by. Her final year dissertation topic also offered her an opportunity to explore aspects of her sexuality. In her first interview, Caitlin showed signs of approaching the analyst’s discourse, becoming energised by her *objet a*.

On occasion, however, Caitlin demonstrated being positioned as a split subject ($), motivated by ambivalent subliminal desires. On the one hand, she had applied for university in the first place because she felt she was missing out on something important. On the other hand, she accepted the consumerist discourse of the university as a commercial enterprise. As a commuting mature student planning her wedding, Caitlin felt she didn’t quite fit in with younger students. The proxy *objet a* provided by the university tessellated with her own perceived sense of lack. When under pressure, she criticised herself for being stupid, too old, and out of place (C1 351–352). Caitlin often positioned herself precariously in the hysteric’s discourse. When the topic of exams came up for example, she embarked on a vividly neurotic narrative about a mysterious psychosomatic skin condition that flared up before her first-year exams. By the time of the second interview a bout of unexplained illness and being pregnant had turned the dissertation into something she was “clawing [her] way through.” Her exhaustion exacerbated her feelings of uncertainty and self-doubt. This was a much wished-for pregnancy, yet it forced Caitlin to retreat into the subsistence agency of university discourse: “I know there’s not much I can do about anything in particular apart from waiting to hear if the pregnancy’s okay and then I know I do need to … just try and focus on the last few assessments and dissertation stuff that I have.” [C2, 103–110].

Like Jack, towards the end of the year Caitlin was experiencing a detachment from her initial desires, necessitated by turbulent personal circumstances. Her disappointment was palpable, but her previous neurotic energy had given way to a calmer resignation to “just get to the end” (C 206). She reflected ruefully that she had derived a greater sense of agency when she was working (C2 189–195) and missed the structure and responsibility of the workplace (C2 186–188). Now her intrasubjective desires had been replaced by the relationality and intersubjectivity of pregnancy; having barely glimpsed the potentialities of subliminal agency, circumstances had propelled her back into subsistence. She felt obliged to settle for less than she knew she was capable of. Instead of situating herself into the analyst’s discourse, Caitlin reluctantly retreated to the predictability and low-risk demands of university discourse. Her relationship with her supervisor remained good but her dissertation’s momentum stalled. She admitted that her attitude towards her studies was “not probably very good at the minute…but yeah, like if you’d said to me last year that I’d be submitting really poor essays this year, or incomplete ones, I’d be like, well I would never do that…it’s literally all you can get out of me at the minute, so there you go >haha<” [C2 141–142]. “I don’t regret coming to uni,” she wistfully concluded, “it’s just that my life has changed so much from when I started to now, so many things have happened, like personally, that have changed my motivations and my feelings towards it that now it’s just – it’s a completely different feeling at the end to how I started.” [C2 375–378].

### Mae’s Journey

Mae’s agency traversed the discourses of the university and the analyst. A mature student who had left a well-paid public service job to go to university, Mae was confident, creative and unconventional, and a joy to have in class. She was not afraid of discussing controversial topics or speaking out against authority. She could embrace university discourse strategically and with ease, whilst confidently speaking from the analyst position. Mae’s strategic engagement with university discourse was informed by her prior work experience, but unlike Caitlin, she was ready and willing to critique and interrogate the university’s manoeuvres while leveraging them for the benefit of her career:

I think [in one of my essays] I tried to make a bit more something I’d be interested in and then it backfired on me. [So] basically they were like ‘You weren’t critical enough.’ It’s like, ‘Well I was, I just wasn’t critical of [what you wanted].’ [M2, 153–165].

Mae’s dissertation played a crucial role in encouraging her to develop her *objet a*. The sustained validation she received from her supervisor boosted her confidence and enabled her to master the challenges of independent research. Her unusual topic allowed her to assimilate her existing interests, skills and experiences with nascent ones, bridging past, present and future identity positions. Her dissertation research was driven by her own curiosity and political values, evidently shaped by her work experiences, and conducted in service of the career she intended to make for herself. Not satisfied by the intangibility of academic pursuits, Mae had sublime goals in mind: “I want someone to be like, ‘Dr. [Mae Smith] saved my kid’s life!’ Like, do you know what I mean? I don’t want someone to be like, hmm, ‘She came up with this theory’. It’s like – that’s not what I want, I want to actually help people who are there in front of me.” (M2, 324–336).

Mae’s one reported hysterical incident came when she got a poor grade on one of her final assignments, and her dogged determination to get a First suddenly felt jeopardised. Her confidence, unshakeable in the first interview, was knocked by this event. While significantly troubled by the grade, Mae nevertheless resiliently attributed her disappointment to be a function of the dominating nature of university discourse. She adopted the analyst’s critical approach: “It’s just because it’s been such a big chunk of my life it’s hard to get your head around … I still will be gutted if I don’t get a First, but I need to then be like, it’s okay. Like literally, it’s not the end of the world.” (M2 343–346) In essence, Mae’s Analytical proclivities enabled her to weather the insults of university discourse and, guided by her self-assurance, by the end of the year had secured a conditional offer to undertake a Master’s degree.

### Zach’s Journey

Zach was our most eccentric interviewee. Like Caitlin and Mae, Zach was not a conventional student, but unlike them, he was unable to make this difference work for him. Oscillating between the discourses of the hysteric and the analyst, his interviews were full of contradictions, hyperbole, and obscure allusions. Simultaneously rude and evasive, yet cheeky and beguiling, Zach spoke from the subliminal position of the protesting hysteric. Highly cynical and ambivalent in his attitudes, he considered the bureaucratic dictates of university discourse somewhat beneath him. Like his friend Mae, he was a vocal critic of the university’s masked authority. He boasted of a Zen-like calm in the face of academic stress, yet expressed disdain for other students who seemed trapped in university or master’s discourses and whose agency was therefore governed by the Other: “If you’ve picked a topic you didn’t like because someone told you to do it, then who do you blame?,” he mused. “Do you blame them or do you blame yourself for not having a mind of your own?.” (Z1 126–128).

By the time of his second interview, Zach’s already strained relationship with his dissertation supervisor had finally collapsed. He interpreted his supervisor’s extended leave as a hurtful act of profound indifference:

He’s going to leave for a month, I thought ‘Who the fuck authorised that? I’m handing in my dissertation thingy and you’re just getting off for a month?’ And he goes ‘By that point [when I get back] I won’t really be able to help you,’ and I thought, ‘Yeah I know you won’t!’ [Z2 77–81].

Zach’s initial sense of the fluid possibilities of abandonment settled into sullen resentment. He forcefully denied that his supervisor had ever been able to offer expertise and guidance. Jack had retreated into the security of university discourse in an analogous situation but Zach was unable to retract himself in this way. His narrative often referenced childhood events, and he remained remarkably vague about the future. When he did discuss the future, it was not in terms of the enticing possibilities of new experiences, but rather in terms of closure, absence, and defeat. If he acknowledged an *objet a* at all, it was “going to go to wherever the wind takes me” (Z2 269). He was still attached to the deviant, normatively unacceptable goal that he recalled his high school teacher having publicly reprimanded him for. Zach’s previous experience with her, recapitulated with his dissertation supervisor, confirmed his view that attachment only made him vulnerable.

Like Jack, Zach accepted his position as being of little importance to the university: “I’m just another name on the very long list of students,” he said. “I think you’d have to have some sort of grandeur problem somewhere along the line if you thought you were anything more than that.” (Z1 253–259) He responded with cynicism when asked if he would seek help from pastoral support staff: “How much time are they going to give you? Know what I mean? Because you’re going to sit there, chat to them – how’s it going? – you could pour your fucking heart and soul to them and then they’ll just be a bit like … yeah, well, keep going!” (Z2 127–129) Zach was variably arrogant and self-effacing, angry yet wryly amused, confident but uncertain. Although he was evidently capable of exhibiting an analyst’s disinterested insight, he nevertheless tended to habitually regress into the hysteric’s position. Facing the existential crises that plague many students approaching graduation, Zach’s ambivalence, detachment, and unwillingness to conform to any prescribed role led him to muse on the struggle to attain subversive agency:

If you try to be an anarchist, you’ve picked a social role – anarchist is a social role, it’s a loop that you’re never going to get out of, so it is what it is, and as soon as you learn that and accept that, because there’s no other way around it, unless you try and start a fucking one-man revolution – (>haha<) again, that’s a social role! Mister Revolutionist, you know what I mean? You can – again, you can’t get out of it, so it is what it is, you’ve got to pick a role and then play it. But – see, I just want to go where I want to go and see what happens (Z2 248–253).

### Molly’s Journey

Molly could comfortably critique the University’s many failings and was securely competent in the Analyst’s discourse. One of the younger undergraduates on her course, she spoke with great energy and enthusiasm. She had plenty to say and did so eagerly. During our first interview at the start of her final year, Molly was readying herself for a period of intensive study, and by our second interview, towards the end of the year, she was quite exhausted. But she also gained a certain confidence she didn’t have before. Her resilience developed through necessity, and she acquired a perspicaciously critical outlook.

Molly was confident in her critique of the university. She appreciated reflexive, responsive teaching, and refused to confer “automatic respect” upon lecturers by whom she did not feel supported (37–40). She valued lecturers who weren’t “scared of silence” (10–11) – who, when posing a question to the class, hold space for students’ answers to emerge in their own time. When lecturers take a *tabula rasa* approach to teaching, assuming students to be naïve and inexperienced, she feels uncomfortable and patronised (48–53), and much prefers to be given opportunities to self-direct her learning (138). Molly’s engagement with university discourse was knowing and tactical. While she resented “jumping through all these hoops to please my marker” (125–126) she recognised that following the rules was necessary for academic success. Her frustration with regulations prompted her to interrogate the effectiveness of higher education, rather than doubt her own abilities, and she developed a perspective that was alternately cynical and hopeful:

Obviously university’s a great thing, you come here and you learn, but you don’t just learn, like you learn how to fit into box and you learn how to double space, and that’s something I’d never thought I need to know but apparently I do … I have a bit of a dim view of university. But I think that’s just because I’ve kind of been enlightened to the way that it could be, but it’s not.” (265–269).

To illustrate the development of her critical streak, Molly cited a “painful” (183) clinical psychology module assignment in which she felt obliged to positively evaluate the efficacy of pharmacotherapy, despite her reservations about its efficacy which were drawn from her personal experiences. To learn about contentious topics, Molly would much rather “sit in a classroom like [Critical and Historical Issues in Psychology] and debate it” (193–194) than copy and repeat “regurgitate[d]” (194) information:

You look at, maybe, society’s norms and you think – is it weird to think that? Or is it normal to think that, and society thinks it’s weird? You know? … Going in every week and just arguing about everything, debating about everything, I think it’s allowed me to kind of challenge everything else in maybe a more articulate way, instead of just getting a bit pent up about it. (218–222).

At this point, the interview slipped into a discussion about mental health, illness, and normalcy, which effectively signified Molly’s struggle for agency and self-determination. Regarding the medical model of mental healthcare, Molly believed that “just because someone thinks in a different way or might deal with things in a different way … you might need a little bit more support, but that doesn’t mean it’s wrong” (322–324). When talking about her own dissertation research into mental health stigma, she transformed from an uncertain undergraduate into a firebrand. Her experiences of friends and family being let down by the mental healthcare system are her motivation, and the alignment of her scholarship with her *objet a* has powerful results. When Molly was diagnosed with a mental health condition, she feared that she was “wrong, because the doctors told me that I need to be fixed” (325–326) – but she now asserts: “I’m not broken” (332). This is highly significant: Molly refused to internalise the implications of the deficit models employed in both higher education and clinical psychology, and thus emerged from these interactions defiant and undiminished. Reflecting on her hopes for the future, she was still weighing up her options, but she did so equipped with a new skill:

I’m able to … take a step back and be like, okay – you’re acting like that because you think you’re the master, and I’m able to look at things with a little more perspective … I’m able to sort of go past that and be like, no, I’m going to do it like this and it’s still going to be okay.” (418–422).

## Discussion

The student journey cannot be operationalised in terms of the gradual acquisition of mere cognitive skills. Not only is the journey replete with hazards, dead-ends and reversals, but it is also a profoundly affective and intersubjective one, saturated with power, desire and potential. Our participants confronted different challenges as they traversed their own journeys. In Lacanian terms, their desire or *objet a* was mobilised or emerged in different ways as they negotiated the four discourses. Whereas Jack’s *objet a* had perhaps always been located beyond the university, focussed as it was on moving from the village to the city and from there into the world, Mae’s *objet a* sustained her academic achievements during her undergraduate degree and looked set to be effective beyond it. Caitlin’s started out as pure academic ambition but altered course when she became pregnant. Zach glimpsed the potential of the analyst’s sublime agency but gravitated towards hysteric’s discourse (“a loop that you’re never going to get out of,” as he put it), unable or reluctant to articulate his *objet a* at this point. Zach was alienated from the learning process, ‘lonely, bored, confused, silenced, fearful of being seen to be stupid, invisible, or much too visible.’ (Mann, 2008, p. 50).

Molly also reflected critically on her student experience and was able to elevate her frustration and disappointment with the institution into a sustained critique. However, she really entered the analyst’s position when our conversation took a tangent towards normalcy in psychology more broadly. Informed by her own lived experience, Molly directly confronted the split subjectivity engendered by normative institutional values when she reflected on her experience of mental healthcare. In her impassioned criticisms of the failures of the system, her education and her emotion worked in tandem, rather than the latter being suppressed in deference to the former. This exemplifies what we have identified as *sublime agency*: the subjective mode produced in the discourse of the analyst, in which the student embodies their *objet a* and works in alignment with their desire. Molly was propelled by a goal much grander than the fulfilment of her degree (although it could still contribute towards it) – a cause that reached beyond the confines of the university. This became Molly’s new master signifier (S1): her desire to effect change, raise consciousness, and empower.

We have argued that of the four discourses, two – the Master and the University – are prioritised but have evident shortcomings in the marketised university. The other two discourses – the Hysteric and the Analyst – may be marginalised but they have expansive potential. There are many opportunities for students to be ‘filled up’ with knowledge (as in Master’s discourse), or to comply with rules and regulations (as in University discourse). These are morphostatic positions of subservience and subsistence, respectively. But there appear to be few opportunities across a rationalised and commercially oriented curriculum for encouraging student morphogenesis by nurturing the *objet a* and shifting from subliminal to sublime agentic positions.

Harris et al. (2018) have reminded educators of their responsibility to design educational opportunities that encourage students to strive towards mastery and growth. We argue that higher education institutions need to treat the market-driven metaphor of the student journey with scepticism and embrace alternative approaches that accommodate the complexities of students’ psychosocial development. It is our hope that our model will facilitate the reflexive questioning of the power dynamics present in the neoliberal university with the aim of nurturing more progressive forms of agency and engagement (Higgins et al., 2019).

## Data Availability Statement

The raw data supporting the conclusions of this article will be made available by the authors, without undue reservation.

## Ethics Statement

The studies involving human participants were reviewed and approved by Manchester Metropolitan University Faculty of Health and Education Ethics Committee. The patients/participants provided their written informed consent to participate in this study.

## Author Contributions

GB and SL co-wrote the manuscript. NF was the principal researcher on the project and also contributed to the writing of the manuscript.

## Conflict of Interest

The authors declare that the research was conducted in the absence of any commercial or financial relationships that could be construed as a potential conflict of interest.

## Publisher’s Note

All claims expressed in this article are solely those of the authors and do not necessarily represent those of their affiliated organizations, or those of the publisher, the editors and the reviewers. Any product that may be evaluated in this article, or claim that may be made by its manufacturer, is not guaranteed or endorsed by the publisher.

## Footnotes

1 The Office for Students, a non-departmental public body of the United Kingdom Government’s Department for Education, is the regulator and competition authority for the higher education sector in England. ‘National Student Survey – NSS,’ https://www.officeforstudents.org.uk/advice-and-guidance/student-information-and-data/national-student-survey-nss/; ‘What is the TEF?’, https://www.officeforstudents.org.uk/advice-and-guidance/teaching/what-is-the-tef/, accessed 29.11.19.

2 Describing the experience of becoming a parent as ‘joyful’ for example, doesn’t adequately capture the phenomenology of the lived experience: there is something missing in the descriptor. Recognising this and invoking the desire to communicate the experience as fulsomely as possible generates the *objet petit a.*

3 Each of the four discourses contain the same four signifiers, arranged in sequence relative to each other. The four invariant positions that each signifier can function within are: agent, other, product, and truth. The four mobile signifiers that can occupy these positions are: the master signifier (S1), the chain of knowledge (S2), the split subject ($), and the objet petit a (a). The signifier that occupies the agent’s position gives each discourse its name. The circulation of the four signifiers through the four positions creates the discourses: master, university, hysteric, and analyst (Table 1). What Lacan calls the ‘master’s discourse’ occurs when the master Signifier (S1) is in the *agent’s* position and the chain of knowledge (S2) in the position of the *other*, the split subject ($) is in the position of *truth* and the *objet a* is the *product.* The four signifiers can occupy any one of four positions, (although they follow an sequential order relative to each other, i.e., S1 S2 $ a S1). In terms of pedagogy, for example, whereas the ‘master signifier’ could represent the lecturer, and the ‘chain of knowledge’ could stand in for the university’s assessment regulations, the ‘split subject’ could symbolise a student’s conflicted identity as both scholar and consumer (Bracher, 1994, 2006; Clarke, 2019).

4 In our experience, when it is pointed out to them, final year students instantly recognise the metaphor of the Master-Slave dialectic as capturing a significant aspect of their undergraduate experience.
